# Burying Hatchets into Endemic Diagnoses: Disseminated Blastomycosis from a Potentially Novel Occupational Exposure

**DOI:** 10.3390/tropicalmed8070371

**Published:** 2023-07-18

**Authors:** Kusha Davar, Arthur Jeng, Suzanne Donovan

**Affiliations:** 1 Department of Medicine, Division of Infectious Diseases, University of California Los Angeles, Los Angeles, CA 90095, USA; 2 Department of Medicine, Division of Infectious Diseases, Olive View-University of California Los Angeles, Los Angeles, CA 91342, USA

**Keywords:** blastomycosis, *Blastomyces dermatitidis*, endemic fungi, axe throwing, occupational exposure

## Abstract

Background: Blastomycosis is an endemic fungal disease predominantly observed in the northern regions of North America. It manifests primarily as pulmonary disease but can also involve dissemination to the skin, bones, and genitourinary tract. Detailed Case Description: We describe a case of a patient in Southern California with disseminated blastomycosis following his occupational exposure to decaying wood. The patient was treated with intravenous amphotericin therapy followed by oral itraconazole therapy with full resolution of his symptoms. Conclusions: The patient’s case presentation serves as a reminder regarding Blastomyces infections diagnosed outside of endemic regions and suggests a potential link between blastomycosis and a novel occupational exposure surrounding axe throwing.

## 1. Introduction

Blastomycosis is an endemic fungal disease often manifesting as pulmonary disease; however, it can disseminate to the skin, bones, and genitourinary tract. This may include pulmonary disease with an indolent pneumonia, skin disease manifested as verrucous skin lesions and cold abscesses, bone disease with painless osteomyelitis and chronic draining sinus tracts, and genitourinary disease leading to prostatitis, epididymitis, endometritis, salpingitis, and tubo-ovarian abscesses [1]. Additionally, blastomycosis is pre-sent endemically in North America, notably often bordering areas surrounding the Mississippi, Ohio, and St. Lawrence Rivers as well as the Great Lakes. Particularly, in Canada, blastomycosis is present amongst four Canadian provinces from Saskatchewan to Quebec [1]. In such endemic regions, *Blastomyces* spp. often inhabit an ecologic niche of forested, sandy, and moist soils; decaying vegetation or organic material; and rotting wood located near water sources [1].

While most infections are sporadic, occupational and recreational activities that disrupt the soil such as construction, clearing brush or cutting trees, hunting, canoeing, boating, and fishing have been associated with outbreaks of the disease [1]. Outbreaks of *Blastomyces* infections have been reported throughout the United States, ranging from endemic regions to areas in Colorado, postulated to be related to vector transmission with prairie dog relocation and the soil disturbances related to its relocation [1]. However, undescribed in the literature is the association of blastomycosis with the handling of wood with axe throwing, a leisurely activity often performed in a group setting or sporting event. We present a case report of a young, otherwise healthy male in Southern California diagnosed with disseminated blastomycosis with a constellation of notable physical exam findings in the setting of an occupational exposure surrounding axe throwing.

## 2. Case Report

A 29-year-old man presented to the hospital with a three-month history of non-productive cough and progressively worsening joint pain in his wrists, knees, and ankles. He moved from Toronto, Canada to Los Angeles, California eight months prior to his presentation. He previously was unemployed in Toronto and began working at an axe throwing facility upon moving to Los Angeles; his duties included chopping wood for customers to use. He reported having a single monogamous female partner and no recent interactions with livestock or other animals outside of his pet dog. 

**Figure 1:**
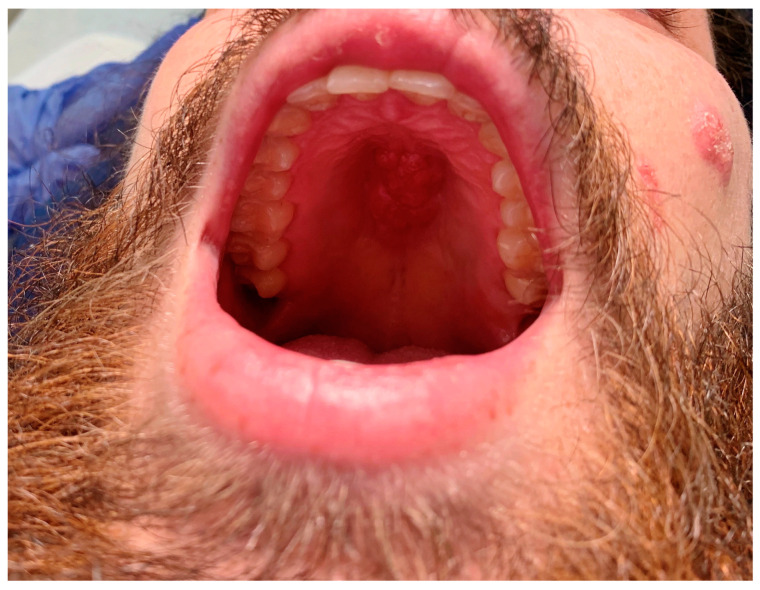
Mass-like lesion present on the hard palate of the patient’s mouth.

His initial presenting symptom several months prior was the presence a cyst in his left lower abdomen which eventually evolved into an abscess with purulent drainage. Several weeks later, he developed a verrucous lesion on the left of his face near his nasolabial fold, followed by other similar lesions throughout his face over the next several months (Figure 1). Gradually, he began having a worsening non-productive cough leading to fatigue and dyspnea on exertion. He denied the presence of fevers throughout its course. Upon examination, he was found to have edema in both hands, knees, and ankles; a soft, fluctuant mass in his right occipital region; and a fixed, hard mass across the roof of his mouth (Figure 1), as well as marked cachexia and temporal wasting. The absence of fevers along with the presence of joint swelling without erythema or warmth pointed towards a more indolent infectious etiology.

The lab work was significant for an initial serum white blood cell count (WBC) of 15.5 K/mL (4.5–10.0 K/mL), hemoglobin count of 10.9 g/dL (13.5–16.5 g/dL), and platelet count of 627 K/mL (160–360 K/mL). The serum creatinine level was 0.58 mg/dL (0.50–1.20 mg/dL), and the liver function test (LFT) results were within normal limits. The high sensitivity C-reactive protein level was 264.5 mg/L (0.0–7.0 mg/L) and the erythrocyte sedimentation rate was 77 mm/hr (no reference range). Notably, fourth-generation HIV Ag/Ab testing was negative.

**Figure 2:**
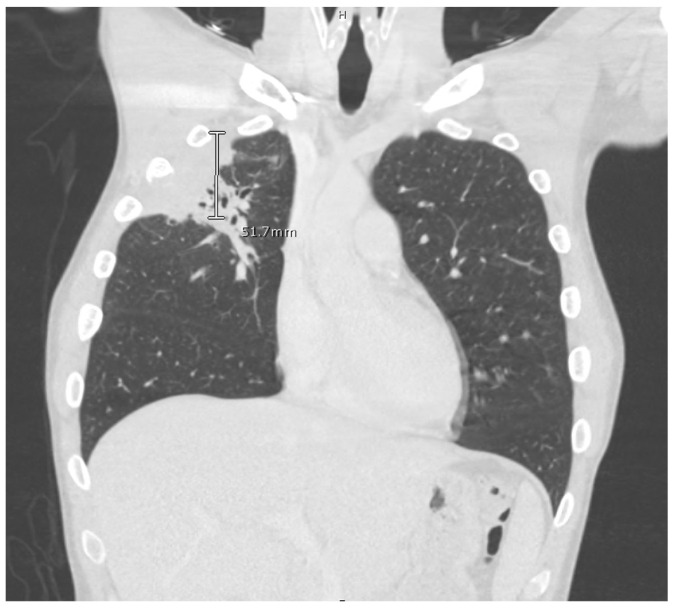
CT scan of chest, right upper lobe pleural and subpleural consolidation with erosive changes at the 3rd and 4th ribs and involvement with the chest wall.

**Figure 3:**
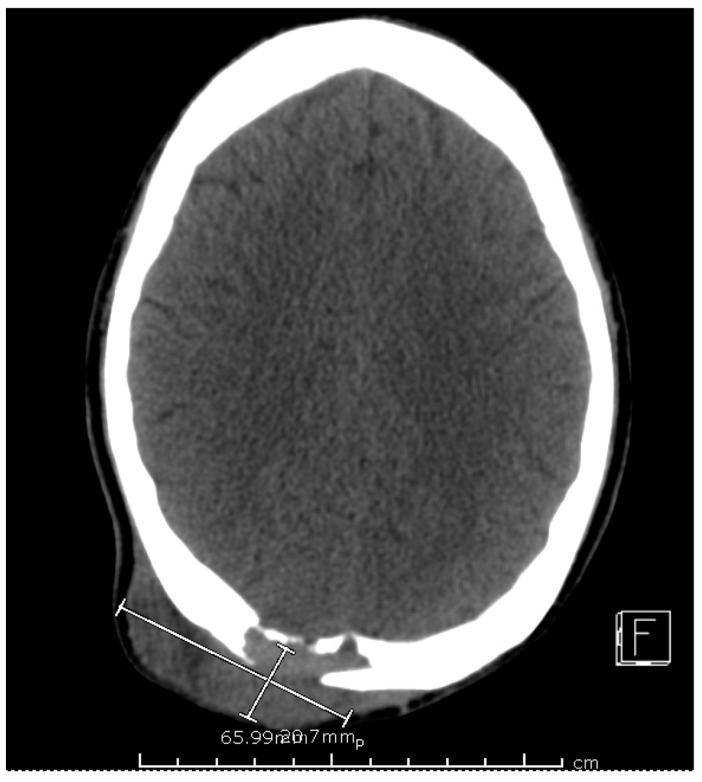
CT scan of head, aggressive lytic lesion of the posterior right parietal bone with an associated 6.6 × 2.1 × 7.1 cm soft tissue mass of the posterior scalp.

The computed tomography (CT) scan of his chest and abdomen revealed a right upper lobe cavitary lesion abutting the ribs (Figure 2) and a sinus tract from his left ischium to the skin over his left lower abdomen, respectively. Additionally, a CT scan of his head was also obtained, which demonstrated a mass extending from his right occipital skull with evidence of skull erosion as well (Figure 3).

Given the CT head scan findings, a lumbar puncture was performed for cerebrospinal fluid (CSF) analysis which showed CSF WBC 0/mL (0–9/mL), RBC 0/mL (0/mL), glucose 66 mg/dl (40–70 mg/dL), and protein 28 mg/dL (15–45 mg/dL). The CSF bacterial, fungal, and ac-id-fast bacilli (AFB) cultures were negative. A synovial fluid analysis of his right knee was also taken which was described as a cloudy fluid demonstrating an RBC count of 151/mL (no reference range); WBC count of 4242/mL (0–199/mL) with 87% neutrophils, 9% lymphocytes, and 4% monocytes; and no crystals present. The bacterial, fungal, and AFB cultures of the synovial fluid of the knee were negative as well.

**Figure 4:**
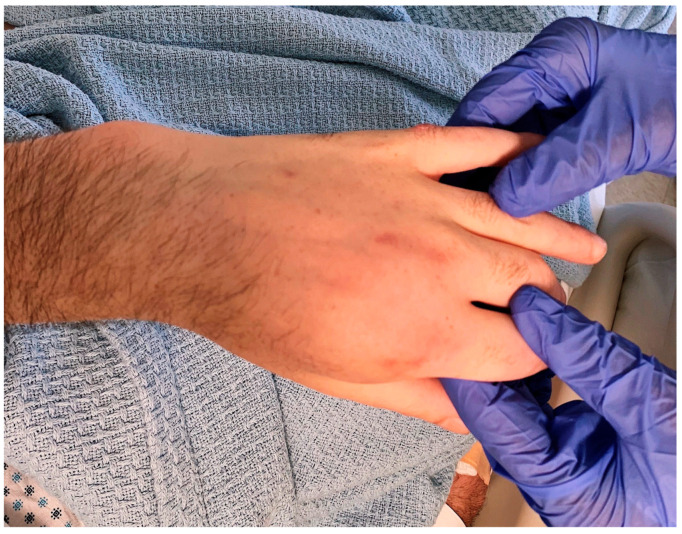
Fluctuant, non-erythematous mass overlying the left 2nd metacarpophalangeal (MCP) joint.

**Figure 5:**
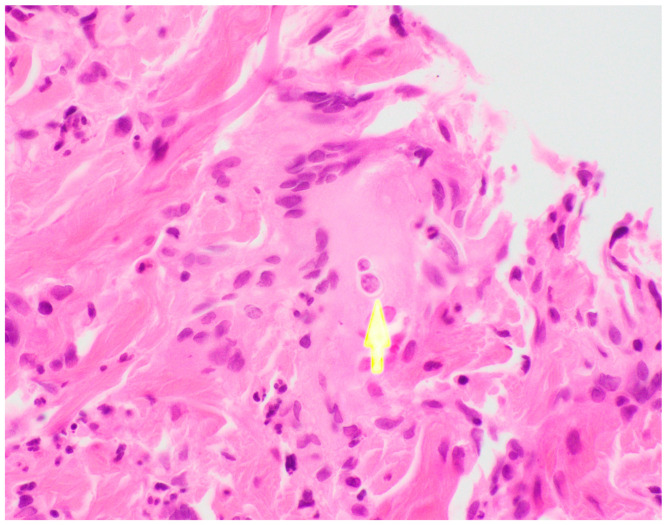
Left 2nd MCP biopsy, H&E stain demonstrating characteristic broad-based budding seen in Blastomyces infections.

Ultimately, a punch biopsy of his left second metacarpophalangeal joint was taken in addition to cultures from the left hip sinus tract drainage which revealed the presence and growth of *Blastomyces dermatitidis* after 12 days of hospitalization, respectively (Figure 4 and Figure 5). Serology testing was also ordered, which was positive for *Blastomyces* antibody immunodiffusion (ID), though negative for Blastomyces antibody complement fixation (CF). Notably, a urine histoplasma antigen test was positive with a result of >25.0 ng/mL with a negative histoplasma antibody ID test, likely indicating a component of cross-reactivity in the presence of *Blastomyces dermatitidis*.

In light of the diagnosis, treatment with liposomal amphotericin was initiated for two weeks, with improvement in the patient’s lesions and respiratory symptoms. Upon completion of his two weeks of intravenous therapy, he was started on oral itraconazole treatment. He followed up several months after the initial diagnosis in the infectious disease clinic while on oral itraconazole, with full clinical recovery and normalization of his cutaneous, mucosal, and joint findings. Ultimately, he was treated with a full twelve months of itraconazole therapy with appropriate serum therapeutic dosing levels until the discontinuation of therapy.

## 3. Discussion

Our case highlights several important clinical aspects surrounding blastomycosis. For instance, the patient presented with an indolent, disseminated disease which illustrates the full spectrum of characteristic findings seen with the disease [1]. However, his presentation also included mucosal findings with a fixed mass on his hard palate which improved with targeted antifungal therapy. Mucosal lesions are infrequently described in the literature, as they are uncommon with *Blastomyces* infections [2]. The indolent nature of *Blastomyces* infections and non-specific clinical manifestations often lead to a delayed diagnosis, particularly when it is not considered on the differential diagnosis in a non-endemic region and when an atypical presentation may be present.

The diagnosis of blastomycosis may be difficult even with a high index of clinical suspicion [1]. For instance, *Blastomyces* in household pets, such a as dogs, can suggest a common source of exposure that may predict human infection [1]. Outside of objective, radiographic data, the diagnosis of blastomycosis can also be confirmed via culture and microscopy [1]. Additionally, serology may be helpful to support a diagnosis, though it is often encountered in the context of variable performance. In our case, the *Blastomyces* antibody ID was positive, and the antibody CF was negative. However, given the microscopic, pathologic results consistent with Blastomyces and the growth of *Blastomyces dermatitidis* in culture, a definitive diagnosis had been established.

Cross-reactivity may be seen with histoplasma antigen assays in the presence of alternate endemic mycoses [3]. Particularly in this case, the patient may have had an endemic risk factor for histoplasmosis prior to living in Los Angeles, though it is not highly endemic geographically [4]. In our case, the histoplasma antigen was markedly positive, which may raise confusion for an alternate diagnosis. However, the presence and growth of *Blastomyces dermatitidis* helped to determine the ultimate etiology of the patient’s presentation. Notably, treatment for disseminated blastomycosis may be different in terms of a recommended regimen for intravenous amphotericin, but fortunately, the diagnosis was achieved to offer the patient optimal therapy.

Initially, upon arrival to the hospital, the initial concern given the patient’s cachectic appearance was a concomitant immunocompromising condition. However, outside of the behavior risk factors, there are no frequently described cases where HIV/AIDS as an immunocompromising condition plays a role in the acquisition of blastomycosis and its dissemination [5]. In sampling hospital admissions for patients diagnosed with blastomycosis, a concomitant diagnosis of HIV/AIDS is seen in approximately 5% of cases [5]. Our patient was sexually active in a monogamous relationship, and his HIV testing was negative, lowering the suspicion of an opportunistic infection from AIDS that may infrequently be seen with blastomycosis and more frequently be seen with other endemic mycoses such as histoplasmosis [6]. 

There is an average incubation period of approximately three weeks to three and a half months for *Blastomyces* infections [7]. In our case, the patient moved from Toronto to Los Angeles eight months prior to his presentation and began handling wood at work imported from the northern regions of North America, presumably contracting his infection in a non-endemic region. This raises the suspicion for an occupational exposure, irrespective of his history of residing in an endemic region in Canada prior to moving to Los Angeles. The patient reported that the wood related to axe throwing was imported from various regions in North America and would often sit out in rainy weather, likely as decaying, moist, wood, before being curated and prepared for axe throwing [1]. Taken into account, the patient’s incubation period exceeds the described incubation period characteristic of blastomycosis, suggesting a unique circumstance for the inoculation of the disease.

Another highlight of this case is the fact that the diagnosis was made in Los Angeles, California. Blastomycosis is a disease seen predominantly in North America, namely, in the northern region of the continent [8]. While *Blastomyces* is endemic to such a region, approximately 8% of hospitalization for *Blastomyces* infections occur outside of endemic regions. However, within this context, California is a rare region for such a hospitalization to occur [5]. In our case, not only was the case diagnosed in Los Angeles, but the patient is assumed to have contracted the infection in Los Angeles as well due to an occupational exposure. Such a diagnosis may be difficult for local providers to establish given the lack of experience with this particular pathogen causing endemic mycosis. To date, outbreaks have been reported throughout various states in the United States, but no outbreak has been reported in California [5,7]. While our case may not represent an outbreak, the potential based on exposure risk may be a harbinger for a potential future outbreak in a non-endemic region.

Lastly, the occupational exposure in this case represents a new risk factor. There is an undescribed correlation between the occupation and such an infection both in the medical literature and the lay population; this may implicate occupational hazards around axe throwing and its wood handling as an emerging occupational exposure that may be linked to blastomycosis. Axe throwing is an age-old activity that has gained popularity in Canada for nearly a decade and has now expanded to both a sporting and leisurely activity on an international level [9]. In communities throughout the United States, axe throwing is an opportunity for people to gather and partake in the sport both in the capacity of an organized league and social gathering [9]. As axe throwing becomes more popular, it will likely expand beyond the North American region to other parts of the world. Certainly, an important role around the risks of infection with axe throwing is the process of wood handling. As wood is often exported throughout different regions of the country, this profession involves both workers and consumers becoming more exposed to several types of wood and the endemic fungi that are regionally linked to the wood. With this activity gaining increased popularity internationally, the risks should be acknowledged to safely handle the wood and materials. This should prompt providers to become aware of the risk factor of this sport and to appropriately raise suspicion in the correct clinical context to screen for this activity in patients.

## 4. Conclusions

This case represents the full clinical syndrome of disseminated blastomycosis with a significant occupational exposure, as the patient reported handled decaying wood as part of his job at an axe throwing facility. Risk factors related to the sport rely on the handling of and exposure to wood, which may serve as a vector for the transmission of endemic mycoses outside of regions classically thought to be endemic. As the sport gains greater popularity worldwide, attention should be brought via possible surveillance to help determine if increased cases and outbreaks are being observed. On the level of the provider, it should be explored moving forward to determine the risks of workers and consumers who may be exposed to *Blastomyces* in addition to being aware of the diagnoses outside of endemic regions, both nationally and internationally.

## Data Availability

Not applicable.
